# The Short Isoform of the Ubiquitin Ligase NEDD4L Is a CREB Target Gene in Hepatocytes

**DOI:** 10.1371/journal.pone.0078522

**Published:** 2013-10-17

**Authors:** Jingqi Fu, Dmitry Akhmedov, Rebecca Berdeaux

**Affiliations:** 1 Department of Integrative Biology and Pharmacology, University of Texas Health Science Center at Houston, Houston, Texas, United States of America; 2 Graduate School of Biomedical Sciences, University of Texas Health Science Center at Houston, Houston, Texas, United States of America; University of Kansas School of Medicine, United States of America

## Abstract

During cycles of fasting and feeding, liver function is regulated by both transcriptional and post-translational events. Regulated protein degradation has recently emerged as a key mechanism to control abundance of specific hepatic proteins under different nutritional conditions. As glucagon signaling through cAMP and PKA is central to glucose output during fasting, we hypothesized that this signaling pathway may also regulate ubiquitin ligases in the fasted state. Here we show that fasting stimuli promote expression of the short isoform of the E3 ubiquitin ligase *Nedd4l* in primary mouse hepatocytes. *Nedd4l-short* mRNA and NEDD4L (short isoform) protein accumulate in glucagon-treated primary mouse hepatocytes and in liver tissues during fasting. We identified a functional cAMP response element in the alternate *Nedd4l-short* promoter; mutation of this element blunts cAMP-induced expression of a *Nedd4l* reporter construct. CREB occupies the endogenous *Nedd4l* locus near this element. CREB and its co-activator CRTC2, both activated by fasting stimuli, contribute to glucagon-stimulated *Nedd4l-short* expression in primary hepatocytes. siRNA-mediated *Nedd4l* depletion in primary hepatocytes did not affect gluconeogenic gene expression, glucose output or glycogen synthesis. Our findings reveal a new mechanism of *Nedd4l* transcriptional regulation in liver cells.

## Introduction

Intermittent food availability requires mammals to store nutrients after feeding and to liberate stored nutrients during fasting. The liver is a major organ responsible for maintenance of normoglycemia and energy balance during cycles of fasting and feeding. In the fasted state, glucagon and catecholamines prevent hypoglycemia by stimulating hepatic glycogen breakdown and gluconeogenesis, in part via the cAMP response element binding protein (CREB) and its co-activator CRTC2 (CREB-regulated transcription coactivator 2) [1]. CREB/ CRTC2 directly and indirectly not only stimulate hepatic gluconeogenesis by transcriptional induction of *Pepck* (encoding phosphoenolpyruvate carboxykinase)*, G6pase* (encoding glucose 6-phosphatase) and *Pgc1α* (*Ppargc1a*, encoding PPAR-gamma coactivator 1-alpha, PGC1α) [2,3], but also inhibit *de novo* lipogenesis [4] and exert a priming effect on post-prandial hepatic insulin sensitivity by transcriptional induction of *Irs2* (encoding Insulin Receptor Substrate 2) [5]. There are more than 4,000 predicted CREB binding sites in the mouse genome [6], so identification of additional CREB/ CRTC2 target genes could shed light on new mechanisms by which these transcriptional activators exert profound effects on hepatic metabolism.

 In addition to transcriptional regulation of metabolic enzymes and regulatory factors, counter-regulatory hormone (e.g. catecholamines and glucagon) signaling during fasting also results in selective post-translational regulation of protein stability through the ubiquitin-proteasome pathway. For example, during fasting, cyclin C/ Cdk8 complexes phosphorylate and stimulate ubiquitin-dependent degradation of Srepb1-c [7]. Similarly, fasting stimulates the p38 MAP kinase-COP1 complex to ubiquitylate hepatic fatty acid synthase (FASN), leading to degradation by the proteasome [8]. In *C. elegans*, fasting induces genes encoding thirty-two different SCF (Skp1-cullen-F box) E3 ligase complex components [9]. Moreover, fasting stimuli induce transcription of the ubiquitin-specific protease *Usp2* in mammalian liver, which contributes to hepatic gluconeogenesis [10]. Finally, the CREB co-activator CRTC2 is targeted for ubiquitin-dependent degradation during late fasting in liver by the E3 ubiquitin ligase COP1 [11]. Thus, signal-induced ubiquitin-dependent degradation, either via transcriptional regulation of E3 ligases or phosphorylation-dependent assembly of E3-substrate complexes, is an emerging mechanism for dynamic control of hepatic metabolism.

 We hypothesized that additional E3 ubiquitin ligases may contribute to regulation of hepatic metabolism during fasting and feeding in the liver. Because the cAMP-PKA pathway is a major intracellular signaling pathway that regulates hepatocyte responses to fasting, we sought E3 ubiquitin ligases expressed in liver that are known to be regulated by cAMP signaling. The HECT family E3 ubiquitin ligase NEDD4L (also called NEDD4-2) is expressed in liver [12] and is acutely inhibited by direct PKA phosphorylation in epithelial cells treated with vasopressin [13]. *In vivo* roles of NEDD4L in liver have not been examined, but NEDD4L is best known for inhibition of the epithelial sodium channel (ENaC) in the kidney [14]. Loss of *Nedd4l* function results in sodium-sensitive hypertension, either due to de-repression of ENaC and other ion channels in the kidney [15,16] or in the brain [17]. *Nedd4l* is also expressed in lung, where it is required for clearance of fluid [18]. Three recent population-based studies found human *NEDD4L* variants associated with type 2 diabetes, obesity and diabetic nephropathy [19–21]. In this study, we identified an unexpected role for cAMP-PKA signaling in regulation of a specific isoform of *Nedd4l* in hepatocytes and liver tissue. We explored transcriptional regulation of this gene by CREB/ CRTC2 and possible roles of NEDD4L in regulation of glucose metabolism in primary mouse hepatocytes.

## Results

### The Nedd4l-short isoform is induced by fasting stimuli in hepatocytes and liver

**Figure 1:**
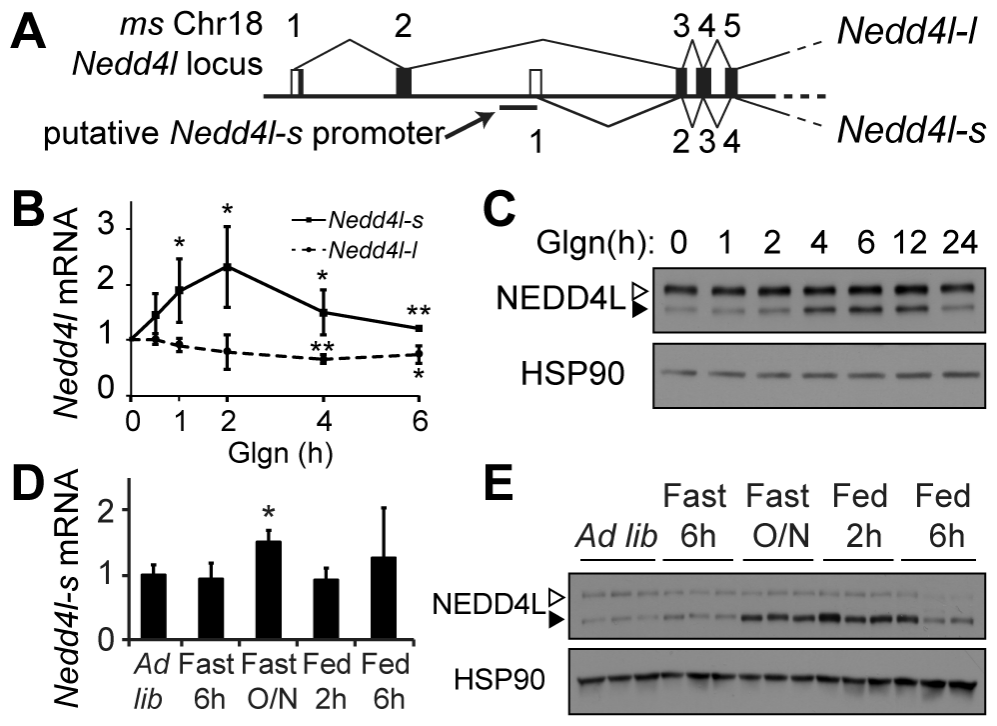
*Nedd4l* short isoform is induced by glucagon in primary hepatocytes and during fasting in mouse liver. (A) Diagram of the first several exons of the mouse *Nedd4l* locus on chromosome 18. Exons incorporated into *Nedd4-long* and *Nedd4l-short* transcripts are indicated by connected lines and numbered above and below the DNA, respectively. The putative alternate promoter for *Nedd4l-short* is shown. Only the first several exons are shown; the rest are shared by the two transcripts. (B) Primary mouse hepatocytes were treated with glucagon (100nM) for the indicated time (h) for analysis of *Nedd4l* isoform mRNA expression. mRNA amounts are normalized to *Gapdh*, represented as mean fold change from 0 h treatment ±stdev. *p<0.05, **p<0.01 to 0 h. (C) NEDD4L proteins and HSP90 loading control in primary hepatocytes treated as in (B). (D) *Nedd4l-short* mRNA from liver tissue of *ad* *libitum* fed, fasted (6 h or overnight ‘O/N’) or O/N fasted and re-fed (2 and 6 h) male C57Bl6/J mice (n=3 per condition). Mean fold change to *ad* *lib*, *p<0.05 to *ad* *lib*, 6 h fasted and 2h re-red. All other combinations were not significant. (E) NEDD4L proteins and HSP90 from liver tissue as in (D). Filled arrowheads, NEDD4L-short; open arrowheads, NEDD4L-long. See Figure S1A, S1D for quantification of western blots.

To identify PKA-sensitive ubiquitin ligases that may contribute to hepatocyte responses during fasting, we searched the literature for E3 ligases regulated by cAMP signaling that are expressed in liver. NEDD4L (Neural precursor cell expressed, developmentally down-regulated gene 4-like, also known as NEDD4-2) satisfied both of these criteria, but nothing was known about the possible roles of this protein in liver. To begin to characterize regulation of NEDD4L by PKA signaling in liver cells, we tested expression of mouse NEDD4L in primary hepatocytes treated with glucagon, a potent activator of cAMP-PKA signaling in this cell type [1]. There are two isoforms of NEDD4L (a short isoform of 110 kDa and a long isoform of 130 kDa), which are encoded by separate transcripts of the same gene (Ensembl v71 [22], Figure 1A; see also *Methods*). mRNA and protein of both isoforms are present in unstimulated primary mouse hepatocytes (Figure 1B, C). We observed that both the mRNA and protein of the NEDD4L short isoform were selectively induced by glucagon within 1 and 4 hours, respectively (Figure 1B, C, S1A). The *Nedd4l-long* isoform (*Nedd4l-l*) mRNA and protein amounts remained unchanged (Figure 1B, C, S1A). Both *Nedd4l-short* mRNA and protein returned to baseline levels after longer treatment, similar to the mRNA pattern for known glucagon-responsive genes *Pepck* and *Pgc1α* (Figure S1B). NEDD4L short isoform (110 kDa) protein induction in hepatocytes was also sensitive to the dose of glucagon, whereas the NEDD4L long isoform protein (130kDa) remained unchanged from control (Figure S1C). 

During fasting, glucagon released by the α-cells of the pancreas stimulates cAMP-PKA signaling. To test whether *Nedd4l* is regulated by fasting *in vivo*, we compared mRNA and protein levels of *Nedd4l* short and long isoforms in mouse liver after fasting and re-feeding. Consistent with our findings in primary hepatocytes, *Nedd4l*-*short* (*Nedd4l-s*) mRNA was also induced in liver tissue after an overnight fast, and the expression returned to control levels following 2 to 6 hours of re-feeding (Figure 1D). Similarly, NEDD4L short isoform protein levels were induced fourfold after overnight fasting and persisted for at least 2 hours after re-feeding (Figure 1E, S1D). By 6 hours after re-feeding, NEDD4L-short protein abundance began to decline, but did not yet return to *ad libitum* levels (Figure 1E, S1D). The effects of fasting on *Nedd4l* expression in liver were specific to the short isoform; there was no significant change in NEDD4L long isoform protein with fasting and re-feeding *in vivo* (Figure 1E, S1D). These data show that the short isoform of *Nedd4l* is selectively regulated by fasting stimuli in hepatocytes*.*

There is a paucity of information about the functional or regulatory differences between the long and short NEDD4L isoforms. Both isoforms contain the substrate-targeting WW domains, but the short isoform lacks the N-terminal C2 domain, which is required for calcium-stimulated plasma membrane targeting in epithelial cells [23]. More recently, calcium was shown to release an inhibitory C2-HECT domain interaction [24]. Although the NEDD4L-short isoform is predicted to lack the N-terminal C2 domain, it has not been reported to have differential substrate selectivity or subcellular localization from the long isoform. In primary hepatocytes, both isoforms were localized to the cytosolic/ membrane fraction and were excluded from the nucleus, irrespective of glucagon treatment (Figure S1E). The functional difference between these two isoforms remains to be determined.

### The short isoform of Nedd4l is a CREB target gene

The rapid and acute regulation of *Nedd4l-s* mRNA by glucagon signaling is consistent with kinetics of CREB/ CRTC2 activity [1] and known CREB target gene (*Pepck, Pgc1α*) induction in hepatocytes, so we investigated whether *Nedd4l* may be a CREB target gene. The two known mouse *Nedd4l* transcripts (each encoding a distinct isoform, short or long) are transcribed with alternate first exons from the same gene and share most exons (Ensembl v71 [22], Figure 1A; see also *Methods*). To determine if *Nedd4l-short* is regulated by CREB, we queried the publicly available CREB target gene database [6] for predicted cAMP response elements (CRE) in or near the *Nedd4l* locus. We noted that two consensus half CRE sites are present in the putative proximal promoter region of the short *Nedd4l* transcript (Figure 1A). We therefore hypothesized that *Nedd4l*-*short* is directly regulated by CREB in glucagon-treated hepatocytes. 

**Figure 2:**
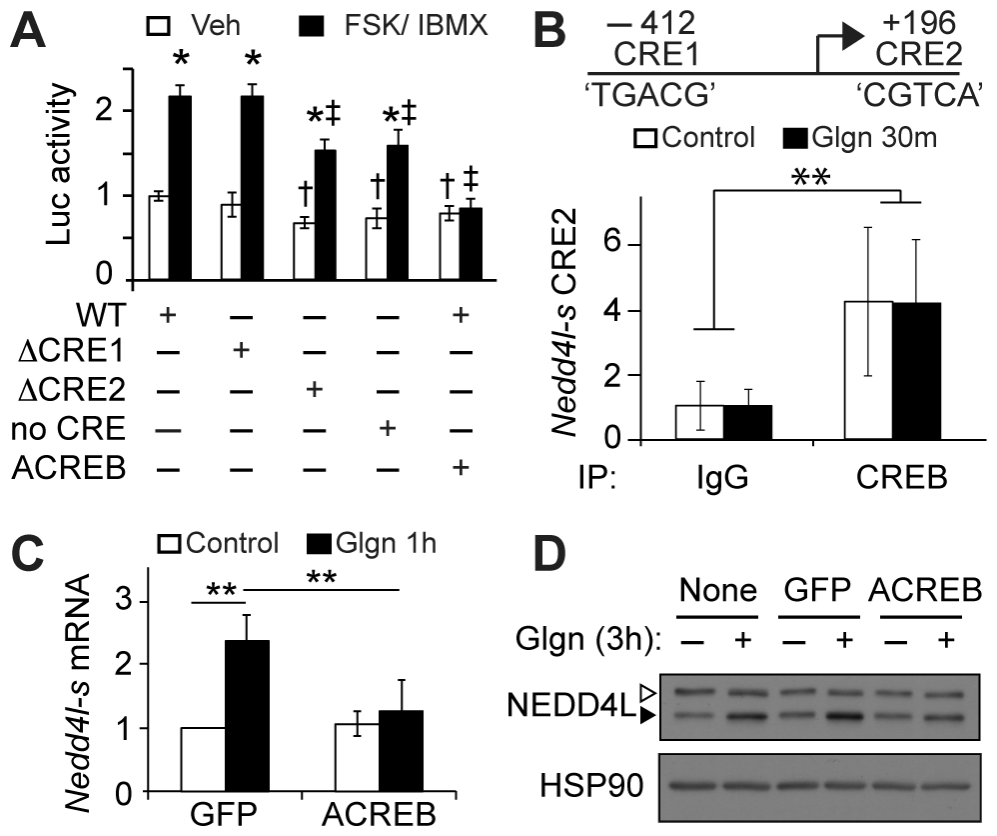
*Nedd4l*-short is a CREB target gene. (A) *Nedd4l-short* luciferase reporter activity in HEK293T cells treated with FSK/ IBMX (6 h). Wild type (WT) *Nedd4l-short* luciferase reporter compared with individual CRE site mutations (ΔCRE1 or ΔCRE2) or both CREs deleted (no CRE). Effect of co-transfected ACREB is shown. *p<0.01 between veh and FSK/ IBMX treated; ‡*p*<0.01 to FSK/ IBMX-treated WT; †*p*<0.01 to veh-treated WT (n=6 replicates shown, representative of three independent experiments). See Figure S2A, B for empty vector control and luciferase data expressed in A.U. (B) Top, predicted CREB binding sites (CRE1: -412 ‘TGACG’; CRE2: +196 ‘CGTCA’) in mouse *Nedd4l-short* promoter. Bottom, chromatin immunoprecipitation from primary mouse hepatocytes treated without or with glucagon (100nM, 30 min) using non-specific IgG (IgG) or anti-CREB IgG. Recovery of *Nedd4l-short* genomic DNA containing CRE2 was quantified in chromatin immunoprecipitates, normalized to the input and expressed as mean fold enrichment ± stdev over matched IgG controls among 3 replicates. **, *p*<0.01 comparing all IgG and all CREB samples. See Figure S2C for *Pepck* and *Gapdh* controls. (n=3 independent experiments) (C and D) *Nedd4l-short* mRNA expression (C, n=4, **p< 0.01) and NEDD4L protein levels (D, n=3) in primary hepatocytes infected with Ad-GFP or Ad-ACREB and treated with glucagon (100nM). Filled arrowheads, NEDD4L-short; open arrowheads, NEDD4L-long. See Figure S2F for quantification of western blots.

To evaluate the contribution of the predicted CREB binding sites to cAMP-stimulated promoter activity, we tested activity of a luciferase reporter encoding the genomic region surrounding the putative *Nedd4l*-*short* promoter (-532 to +321, Figure 2A, 2B top) in HEK-293T cells. This region contains the two consensus CRE sites (CRE1 -412~-407 ‘TGACG’ and CRE2 +196~+201 ‘CGTCA’). Treatment of cells with a cocktail of the adenylyl cyclase agonist forskolin (FSK) and the phosphodiesterase inhibitor IBMX, which induces sustained cAMP production, stimulated *Nedd4l*-*short* luciferase activity but not luciferase activity of the empty vector control (Figure 2A, S2A). We mutated each CRE in the putative *Nedd4l-short* promoter singly and in combination and found that cAMP-stimulated *Nedd4l*-*short* luciferase activity was unaffected by mutation of CRE1. Mutation of the second CRE site (CRE2) resulted in reduced *Nedd4l*-luciferase activity in both vehicle- and FSK/ IBMX-stimulated cells. Mutation of both CRE sites (no CRE) yielded similar luciferase activity to the constructs with mutation of only CRE2 (Figure 2A, S2B). These data show that the first CRE site is likely not functional and the second site accounts for a portion of cAMP-stimulated luciferase activity. To test whether CREB is necessary for *Nedd4l*-luciferase activity, we co-transfected a dominant negative CREB mutant (ACREB) to block CREB activity. ACREB reduced basal *Nedd4l-short* luciferase activity and completely abrogated FSK/ IBMX-stimulated *Nedd4l*-luciferase activity (Figure 2A, S2B). Thus, full cAMP-stimulated induction of the *Nedd4l*-*short* promoter requires the second CREB binding site in the DNA and CREB activity. The fact that dominant-negative CREB completely blocks promoter activity suggests that additional, non-canonical CREB responsive elements exist in the promoter region.

To extend our findings to endogenous *Nedd4l* regulation, we first tested whether CREB associates with CRE2 in the *Nedd4l-short* promoter. We stimulated primary mouse hepatocytes with glucagon for 30 min and performed chromatin immunoprecipitation with unspecific or anti-CREB antiserum (Figure 2B, S2C). The *Nedd4l-short* genomic DNA containing CRE2 was enriched by fourfold in the CREB immunoprecipitates compared to control antiserum. Similar to other CREB target genes, including *Pepck* (Figure S2C), glucagon did not appear to affect CREB occupancy at the *Neddl-short* locus near the CRE2 site. We were unable to detect CREB association with CRE1 (not shown), consistent with the luciferase assay results, and unrelated genomic DNA sequence from the *Gapdh* locus was not recovered in the immunoprecipitates (Figure S2C). We next asked whether CREB is required for glucagon-stimulated *Nedd4l-short* expression in primary hepatocytes. We infected the cells with adenovirus encoding GFP control or ACREB to block CREB activity. We confirmed that ACREB was expressed and blocked induction of *Sik1* (encoding salt inducible kinase 1, SIK1), a known CREB target gene in hepatocytes [2] (Figure S2D, E). In agreement with the luciferase assay data, ACREB inhibited both *Nedd4l-short* mRNA (Figure 2C) and protein (Figure 2D, S2F) induction by glucagon. The *Nedd4l-long* isoform protein was unaffected (Figure 2D, S2F). We observed marginal induction of NEDD4L-short protein in glucagon-treated hepatocytes expressing dominant-negative CREB (Figure 2D, S2F) that failed to reach statistical significance compared to the GFP-expressing controls. It is therefore possible that glucagon stimulates NEDD4L protein accumulation by an additional mechanism, such as a post-translational effect on protein stability. Nonetheless, our data show that endogenous CREB associates with a 150-bp region surrounding the CRE site in the *Nedd4l-short* locus, and CREB activity is required for full induction of *Nedd4l-short* mRNA and protein by glucagon within 1-3 hours.

### The CREB co-activator CRTC2 regulates Nedd4l-short

**Figure 3:**
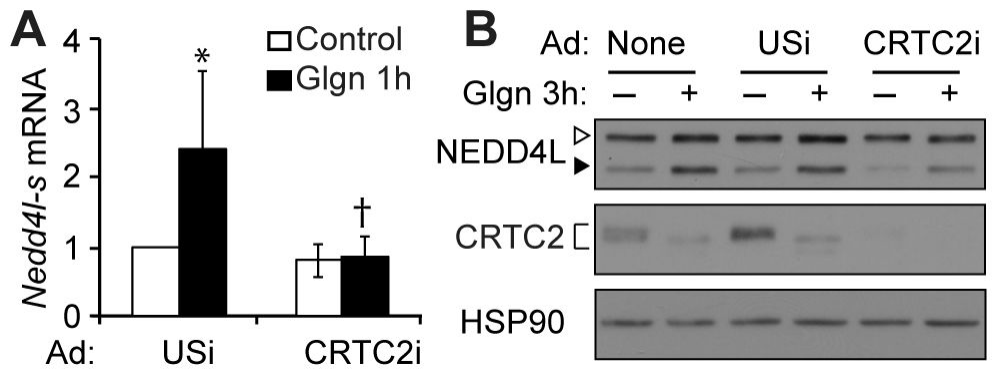
CRTC2 is required for *Nedd4l* short isoform regulation. Primary hepatocytes were infected with adenovirus encoding unspecific shRNA (Ad-USi) or CRTC2-specific shRNA (Ad-CRTC2i) and treated with glucagon (100 nM). (A) *Nedd4l-short* mRNA expression, expressed as mean fold change to GFP control ± stdev (n=4 samples averaged among three independent experiments). *p<0.05 to unstimulated, †*p*<0.05 to glucagon-treated Ad-USi-infected. (B) Western blots of NEDD4L isoforms, CRTC2 and HSP90 loading control (representative of four experiments). Filled arrowheads, NEDD4L-short; open arrowheads, NEDD4L-long; bracket, phospho- and dephospho-CRTC2.

In liver, the CREB co-activator CRTC2 contributes to CREB target gene expression in early and late fasting [2,5,25,26]. To test whether CRTC2 is required for *Nedd4l-short* expression in primary hepatocytes, we infected the cells with adenoviral vectors encoding an unspecific shRNA or CRTC2-specific shRNA (‘CRTC2i’) to knockdown CRTC2 [2]. Similar to our results with ACREB, depletion of CRTC2 blocked *Nedd4l-short* mRNA induction within 1 hour of glucagon treatment (Figure 3A). Similarly, CRTC2 knockdown reduced both basal and glucagon-stimulated NEDD4L-short protein in primary hepatocytes, but some NEDD4L-short protein was still induced by glucagon (Figure 3B); the low expression of NEDD4L-short in CRTC2i-infected cells precludes accurate densitometry. CRTC2 protein was nearly undetectable in the cells infected with Ad-CRTC2i (Figure 3B). In resting hepatocytes, CRTC2 is known to exist in latent cytoplasmic complexes with 14-3-3 proteins. Upon glucagon stimulation, CRTC2 becomes dephosphorylated and moves to the nucleus, where it associates with CREB and CBP [1,2]. As expected, we observed a downshift of CRTC2 protein in glucagon-treated hepatocytes (Figure 3B). The NEDD4L long isoform protein levels were not altered by CRTC2 knockdown (Figure 3B). These data show that CRTC2 is required for the acute induction of *Nedd4l-short* mRNA by glucagon in primary hepatocytes. Taken with our other findings, our results support a model in which CREB and CRTC2 regulate *Nedd4l-short* via CRE or CRE-like elements in the alternate short isoform promoter region.

### Nedd4l is not required for gluconeogenesis, glycogen synthesis or lipogenesis in primary hepatocytes

We have shown that *Nedd4l*-*short* is a CREB target gene that is induced by glucagon in hepatocytes and by fasting in the liver. In other cell types, NEDD4L is known to target several proteins for ubiquitin-dependent degradation or altered localization, including numerous ion channels (ENaC is the major example) [27] and activated Smad2/3 transcription factors [28]. ENaC is not known to be highly expressed in hepatocytes, and we observed no change in Smad2 or Smad3 abundance or turnover after TGF-β treatment in primary hepatocytes transfected with NEDD4L-selective siRNAs (not shown). Recent proteomics analysis showed that human NEDD4L is capable of ubiquitylating more than 100 proteins, most of which contain consensus PPxY recognition motifs [29]. The role of NEDD4L-dependent regulation of many of these targets in cells or tissues is not yet validated, and *in vivo* roles of NEDD4L in hepatic metabolism have not yet been evaluated. 

**Figure 4:**
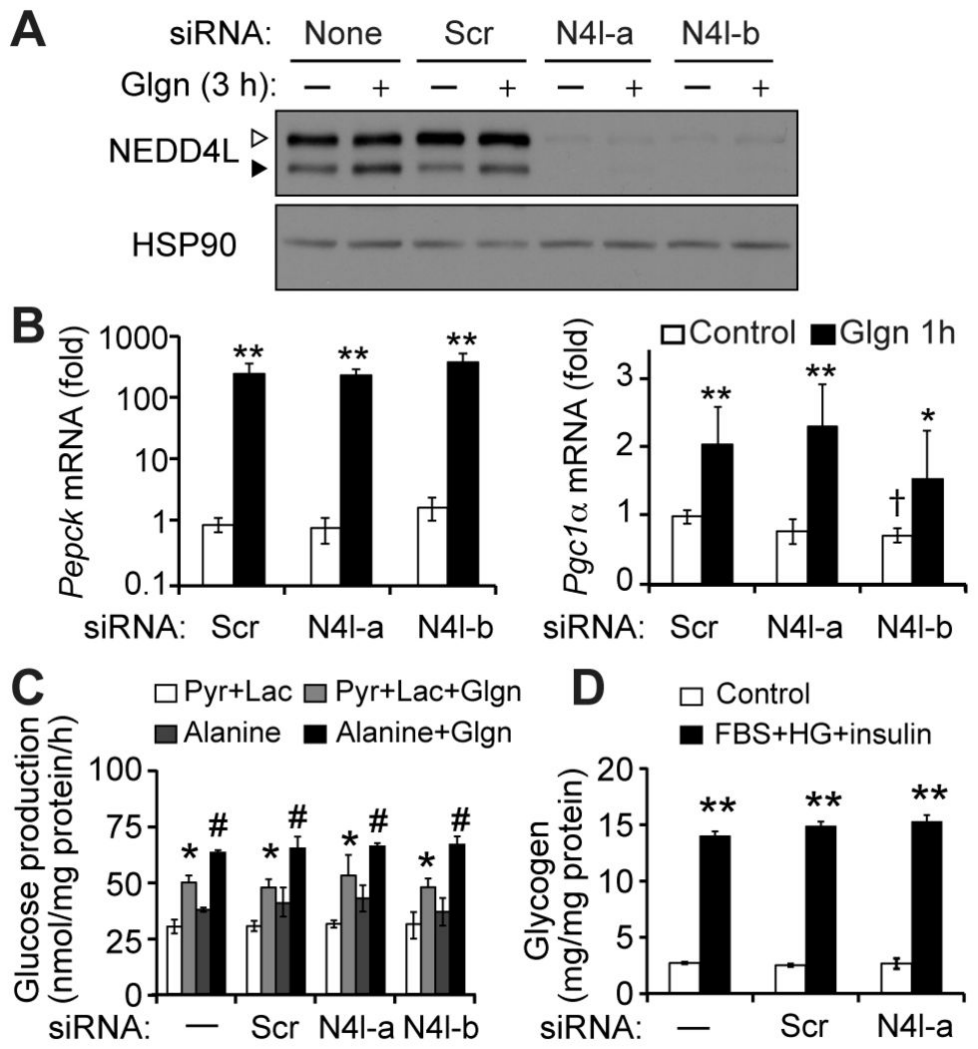
NEDD4L does not affect glucose metabolism in primary hepatocytes. (A) Western blot of NEDD4L isoforms in primary hepatocytes transfected with indicated siRNAs, untreated or treated with glucagon (100nM) for 3 h. Scr: scrambled siRNA control; N4l-a, N4l-b: two different siRNAs specific to *Nedd4l*. Filled arrowheads, NEDD4L-short; open arrowheads, NEDD4L-long. (B) Basal and glucagon-stimulated gluconeogenic gene expression (Pepck, Pgc1α) in control and NEDD4L-deficient hepatocytes. Data expressed as fold of unstimulated Scr ±stdev; n=4-5 replicates among 2-3 independent experiments; **p<0.01, *p<0.05 compared to unstimulated control for each siRNA, †*p*<0.05 compared to scrambled siRNA control. (C) Glucose production from pyruvate + lactate (Pyr+Lac) or alanine in control or siRNA-transfected hepatocytes under basal and glucagon-stimulated conditions. Glucagon had a significant stimulatory effect in all groups; within groups of cells receiving the same siRNA, *p<0.05 compared to Pyr+Lac alone, #*p*<0.05 compared to alanine alone; no significant effect of the *Nedd4l* siRNAs (mean of 3 biological replicates; representative of two independent experiments). (D) Glycogen synthesis in siRNA-transfected primary hepatocytes treated with FBS, high glucose and insulin (FBS+HG+insulin, 3h). No significant effect of the siRNAs; **p<0.01 to unstimulated control. Mean of 3 biological replicates ±stdev; representative of three independent experiments in triplicate.

Based on the striking induction of NEDD4L short isoform abundance in fasted liver and the known roles of CREB/ CRTC2 to regulate hepatic glucose production [1], we hypothesized that NEDD4L may contribute to regulation of glucose metabolism in hepatocytes. We used siRNAs specific to a common region of both *Nedd4l* isoforms to deplete NEDD4L in hepatocytes, as the mRNAs of these two isoforms are nearly identical; any observed effects could subsequently be assigned to the short or long isoform by rescue studies or isoform-selective siRNAs. Transfection of two different *Nedd4l*-specific siRNAs abrogated both basal and glucagon-stimulated NEDD4L protein expression (Figure 4A). We first tested whether NEDD4L affects glucagon-induced expression of mRNAs encoding *Pgc1α* and *Pepck* in primary hepatocytes [30]. As expected, glucagon strongly induced both *Pgc1α* and *Pepck*. Knockdown of NEDD4L did not affect glucagon-stimulated expression of these genes, but one *Nedd4l*-selective siRNA slightly reduced basal *Pgc1α* expression (Figure 4B). Because this reduction was limited to a single siRNA, we do not believe it is a biologically meaningful effect. We further directly tested glucose output from control and *Nedd4l*-deficient hepatocytes under control or glucagon-stimulated conditions. Consistent with expression of gluconeogenic genes, we found that glucagon-stimulated gluconeogenesis from pyruvate and lactate was similar in untransfected hepatocytes and those expressing either unspecific or *Nedd4l-*specific siRNAs (Figure 4C). Because NEDD4L can regulate numerous transporters and ion channels, we also tested whether NEDD4L may be required for glucose production from the amino acid alanine. However, glucose synthesis from alanine was also unaffected by knockdown of NEDD4L (Figure 4C), suggesting that alanine import is unimpaired. Thus, NEDD4L isoforms are not required for glucagon-stimulated gluconeogenesis in primary hepatocytes.

In the post-prandial state, insulin stimulates storage of excess glucose as glycogen and lipid in the liver [31]. During fasting, glucagon-stimulated CREB/ CRTC2 complexes sensitize the liver to postprandial insulin signaling by transcriptional induction of *Irs2* [5]. We therefore hypothesized that NEDD4L may be induced during fasting for a role in insulin signaling or insulin-induced events during the immediate post-prandial period, when NEDD4L protein levels remain elevated (Figure 1E). We observed no change in acute insulin-stimulated Akt phosphorylation in primary hepatocytes expressing *Nedd4l*-selective siRNAs (not shown). To test whether NEDD4L impacts dynamics of glycogen storage in hepatocytes, we treated primary hepatocytes with fetal bovine serum (FBS), high glucose and insulin for 3 hours to stimulate glycogen storage [32]. As expected, insulin treatment dramatically stimulated intracellular glycogen accumulation, but NEDD4L knockdown did not affect insulin-stimulated glycogen storage (Figure 4D), glucagon-stimulated glycogen breakdown after this initial loading period (not shown) or insulin-stimulated lipogenesis (Figure S3). These results show that NEDD4L does not regulate glycogen or lipid synthesis in primary hepatocytes cultured *ex vivo*. 

## Discussion

We set out to identify PKA-regulated ubiquitin ligases expressed in the liver that may impact hepatic metabolism. Previous studies had shown that PKA directly phosphorylates and regulates the HECT domain E3 ligase NEDD4L [13]. We therefore evaluated *Nedd4l* expression in primary mouse hepatocytes and liver tissue. We present the first evidence that the short isoform of *Nedd4l* is regulated by fasting in liver *in vivo* and by glucagon signaling in primary mouse hepatocytes *ex vivo. Nedd4l-short* is transcribed from an alternate promoter within the *Nedd4l* gene. We show that *Nedd4l-short* is a CREB target gene: the regulatory region contains a consensus cAMP response element (CRE) that is necessary for full cAMP-stimulated transcriptional activity and physically associates with endogenous CREB, and *Nedd4l-short* transcription is blocked by dominant-negative CREB or knockdown of CRTC2. Our data clearly indicate that the CREB/ CRTC2 complex is required for acute glucagon-stimulated *Nedd4l-short* mRNA induction in primary hepatocytes and that CREB is required for *Nedd4l-short* promoter luciferase activity. The finding that mutation of the functional CRE site we identified only partially reduced the promoter activity indicates that additional cAMP- and CREB-sensitive elements exist in the sequence. However, we did not identify additional canonical CRE elements in the promoter region we cloned. 

In liver tissue, NEDD4L-short is strongly induced during fasting and declines after re-feeding. Although several studies show that NEDD4L is regulated by post-translational mechanisms in different tissues, there is little known about mechanisms by which the *Nedd4l* mRNA transcription is regulated. Several of the human *NEDD4L* transcripts are androgen sensitive in prostate cancer cell lines [33], but other extracellular cues have not been identified. cAMP-dependent regulation of *Nedd4l* transcription could serve as a mechanism to regulate the abundance or surface expression of specific ion channels or signaling mediators in response to endocrine hormones in liver. Although either expression of dominant-negative CREB or CRTC2-selective shRNA almost entirely blocked acute *Nedd4l-short* transcriptional induction, NEDD4L-short protein still accumulated to some extent in response to glucagon. It is therefore likely that additional mechanisms contribute to the total amount of NEDD4L-short protein expressed after glucagon treatment in hepatocytes. For example, it is possible that cAMP signaling regulates NEDD4L-short protein stability as well as transcription, as we have recently shown for SIK1 [34]. It is also possible that additional transcriptional regulators contribute to *Nedd4l-short* induction during fasting *in vivo*. It is notable in this regard that the gluconeogenic gene *G6pase* is primarily regulated by CREB/ CRTC2 during early fasting; at later times, these complexes are replaced by FOXO-containing transcriptional complexes [11]. *G6pase* promoter constructs lacking CREB binding sites are poorly expressed during early fasting, but expressed at normal levels during late fasting [11]. It is not clear whether this model is generalizable to all CREB- and FOXO-regulated genes in liver. For example, *Irs2* is regulated by CREB and CRTC2 in liver. Depletion of *Crtc2* in liver *in vivo* completely blocks IRS2 expression after overnight fasting despite the existence of consensus FOXO binding sites in the *Irs2* promoter [5]. We did not identify a consensus FOXO binding site in the proximal promoter region of *Nedd4l-short*. In *Crtc2* ^*-/-*^ mice, expression of gluconeogenic mRNAs including *G6pase* and *Pepck* is reduced in liver after overnight fasting [25,26]. *In vivo* studies will reveal the extent to which CREB and CRTC2 are required for *Nedd4l-short* expression during early and late fasting. 

We show that NEDD4L is readily detectable in liver and highly regulated by fasting stimuli, but its physiological role in this tissue remains unknown. Our findings of strong and selective cAMP-CREB dependent regulation of the *Nedd4l-short* mRNA and protein during fasting suggest that the NEDD4L short isoform functions during fasting or in the postprandial state, probably regulating processes other than glucagon-induced glucose output, insulin- and glucagon-regulated glycogen synthesis and degradation, or lipogenesis. Many major metabolic and functional changes occur in the fasted liver, including enhanced ketogenesis, lipid oxidation, and amino acid catabolism, most of which are best studied *in vivo* where analysis can be performed in the context of the other metabolic tissues and full complement of endocrine regulatory hormones. Perinatal lethality precludes analysis of metabolic phenotypes in adult *Nedd4l* knockout mice [18], but a conditional *Nedd4l* knockout model was recently reported [16]. It will be interesting to investigate other possible metabolic roles of NEDD4L isoforms in liver *in vivo* in *Nedd4l* knockout mice to help determine why mutations in human *NEDD4L* loci are associated with risk for type 2 diabetes [19], obesity [20] and diabetic nephropathy [21].

## Methods

### Ethics statement

All animal experiments in this study were approved by Animal Welfare Committee of the University of Texas Health Science Center at Houston (HSC-AWC-11-095) following all current NIH guidelines for animal care and welfare.

### Primary hepatocytes

Primary mouse hepatocytes were isolated from C57Bl6/J mice (Jackson Laboratories) by the modified collagenase method as described [35]. Briefly, mice were anesthetized using isoflurane and the liver was perfused via the descending vena cava with Hank’s balanced salt solution (HBSS, 5.33 mM KCl, 0.44 mM KH_2_PO_4_, 138 mM NaCl, 4.2 mM NaHCO_3_, 0.34 mM Na_2_HPO_4_, 5.6 mM glucose) containing 50 mM HEPES, 5 mM EGTA, pH 7.4, and then with HBSS containing 50 mM HEPES, 5 mM CaCl_2_ and 100 U/mL collagenase type IV (Sigma, C5138) at a rate of 2 ml/min. Livers were removed, and cells were dissociated and washed with HBSS. This method typically yields ~70% viable hepatocytes, which were plated at 1x10^5^ viable cells/cm^2^ in plating medium (M199, 10% FBS (v/v), 2 mM glutamine, 100 nM dexamethasone, 100 U/mL penicillin, and 100 µg/mL streptomycin). After cell attachment (3 h), the medium was replaced by fresh maintenance medium (M199, 2 mM glutamine, 100 U/mL penicillin, 100 µg/mL streptomycin). The day after plating, primary hepatocytes were transfected with 5 nM scrambled unspecific (si-Scr) or *Nedd4l*-specific siRNA duplexes (Sigma, sequences in Table S1) with Lipofectamine 2000 (Life Technologies) or were infected with adenovirus (~20 PFU/cell) encoding GFP (Ad-GFP), ACREB (Ad-ACREB) [3], unspecific shRNA (Ad-USi) or CRTC2-selective shRNA (Ad-CRTC2i) [2], yielding 100% infection based on co-expressed GFP in all viruses. Experiments were performed 24 h after transfection or infection. 

### Animal tissue

Wild-type 8-12 week-old male C57Bl6/J mice maintained on normal chow diet were fed *ad libitum*, fasted for 6 or 16 h (overnight, ‘O/N’), or fasted for 16 h and re-fed with normal chow for 2 or 6 h prior to euthanization by CO_2_ inhalation with immediate removal of the liver tissue. Liver tissue was rinsed quickly in cold 1X PBS, snap frozen in LN_2_, and stored at -80°C until use. For extraction of protein and mRNA, liver tissue was pulverized with a mortar and pestle under LN_2_ and homogenized in the appropriate lysis buffer on ice using a rotor-stator prior to further purification. 

### Plasmids

The *Nedd4l-short* promoter region (-538 to +321) was amplified from mouse genomic DNA by PCR and cloned into the *Hind*III*-Xho*I restriction sites of pXP2 to create *Nedd4l*- luciferase. CRE sites were mutated by quick-change mutagenesis using PfuTurbo (Agilent): CRE1 (-412 to -407 TGACG to CTAGA) and/ or CRE2 (+196 to +201 CGTCA to CATGG). Oligonucleotide primer sequences are listed in Table S1. CMV-driven Flag-ACREB [36] and RSV-β-galactosidase plasmids were gifts of Dr. Marc Montminy.

### Promoter analysis

*Nedd4l* promoter sequences and transcript data were taken from the Ensembl database (v71) using the *Mus musculus* GRCm38 assembly [22]. Transcript *Nedd4l-201* (referred to in this study as *Nedd4l-short* because it is predicted to encode the short protein isoform) initiates from an alternate exon downstream of exon 1 of *Nedd4l-202* (Figure 1A)*. Nedd4l-201* comprises 30 exons, contains 8,212 nucleotides and is predicted to encode an 855 amino acid protein (NEDD4L-short). *Nedd4l-202* (referred to in this study as *Nedd4l-long*) comprises 31 exons, contains 8,163 nucleotides and is predicted to encode a 976 amino acid protein (NEDD4L-long). Promoter sequences were further analyzed using the DNAStar Lasergene suite. *Nedd4l-201* (*Nedd4l-short*) promoter numbering is based on the transcription start site (+1) annotated in the Ensembl v71 assembly.

### Luciferase assays

HEK293T cells (ATCC) were transfected with promoter luciferase expression constructs, Rous sarcoma virus LTR-driven (RSV) β-galactosidase plasmid, and expression constructs (pZeo-ACREB) or empty vector controls with Lipofectamine 2000 for 24 hours. Cells were stimulated with DMSO vehicle or a mixture of forskolin (FSK, 10 µM, EMD) and 1-isobutyl-3-methylxanthine (IBMX, 18 µM, Sigma) for 6 h. Luciferase and β-galactosidase activities were determined as described [37]. Luciferase activity was normalized to β-galactosidase activity, represented as fold change to vehicle-treated, wild type *Nedd4l-short* luciferase control or as arbitrary units (A.U.). 

### Analysis of proteins

Whole cell extracts were prepared from cells and tissues in ice-cold modified RIPA-T buffer, sonicated, clarified by centrifugation and protein concentration determined [34]. For cell fractionation, hepatocytes were washed with cold PBS and lysed in hypotonic lysis buffer (50 mM HEPES pH 7.4, 10 mM NaF, 1 mM EDTA, 0.5% NP-40, 0.25M sucrose, 0.5 mM DTT with protease inhibitors) followed by 30 dounces in a glass homogenizer with a B-type pestle. Nuclei were pelleted (1,200x*g*, 5 min 4°C), washed several times in hypotonic lysis buffer, and lysed in nuclear extraction buffer (50 mM HEPES pH 7.4, 420 mM NaCl, 10 mM NaF, 1 mM EDTA, 0.5 mM DTT with protease inhibitors) followed by homogenization with a plastic pestle. Final fractions were clarified at 14,000x*g*, 30 min 4°C. Extracts were boiled, resolved on SDS-PAGE gels, and then transferred to PVDF membrane for western blot and detection by ECL. Antibodies: anti-NEDD4L (Cell Signaling, 4013), anti-CRTC2 (Epitomics, #3565-1), HSP90 (Santa Cruz, sc-7947). Western blots were quantified by densitometry (ImageJ) on unsaturated films; signals normalized to loading control in arbitrary units, expressed as fold change of control.

### Gene expression

Total RNA was extracted from hepatocytes or liver tissue with on-column DNAse digestion (5 PRIME) and cDNA prepared by MMLV reverse transcriptase (Invitrogen) using a *Nedd4l* gene-specific RT primer and oligo(dT)_20_ primer in the same reaction. To quantify the mRNAs encoding the two *Nedd4l* isoforms, a gene-specific RT primer common to both *Nedd4l* isoforms was used for cDNA production followed by real-time PCR for long or short *Nedd4l* isoforms using exon-specific primers. For *Nedd4l-short*, both forward and reverse primers recognize the first exon, which is absent in the long form. For *Nedd4l-long*, qPCR primers amplify a region encoded by exons 1 and 2 that is absent in the short isoform. Relative mRNA abundance was determined by real-time PCR with SYBR green detection as described [38], normalized to *Gapdh* internal control, expressed as fold change of control averaged over multiple experiments or experimental replicates, as indicated. See Table S1 for primer sequences.

### Chromatin immunoprecipitation

Primary mouse hepatocytes were stimulated for 30 min with vehicle or glucagon (100 nM), crosslinked and neutralized as described [39]. Chromatin in cell lysates was sonicated to an average size of 500 bp using a Covaris S220 UltraSonicator. 1 mg of crosslinked extract was incubated with 2 µg/mL normal rabbit IgG (Cell Signaling #2729) or rabbit anti-CREB IgG (Cell Signaling #9197) at 4°C O/N prior to capture on Protein G Dynabeads (Life Technologies). Beads were washed extensively, crosslinks reversed and protein digested with Proteinase K, and chromatin purified on columns (Zymo Research). Recovery of specific genomic regions (*Nedd4l-short* CRE2 +126 to +271, positive control *Pepck* CRE site -96 to +14, and negative control Gapdh +453 to +620) was determined by quantitative real-time PCR with relative quantification compared with input fractions or by visualization of PCR products on agarose gels.

### Glucose output assay

Glucose output was determined as described [40]. Primary mouse hepatocytes were washed twice with warm PBS and once with glucose-free DMEM. Cells were incubated with phenol red-free, glucose-free DMEM, supplemented with 20 mM sodium lactate + 2 mM sodium pyruvate or 20 mM alanine for 3 h with or without 100 nM glucagon. Glucose concentration in the medium was measured enzymatically [41] by incubation in 150 mM HEPES, 15 mM MgCl_2_, 3 mM EDTA, 2.5 mM NADP, 2.5 mM ATP, 2.5 U/ml glucose-6-phosphate dehydrogenase, 5 U/ml hexokinase for 10 min, room temperature. Glucose concentration was calculated based on A340 relative to a standard curve and normalized to protein content (BCA assay). 

### Glycogen synthesis assay

Glycogen synthesis was determined as described [32,42]. siRNA-transfected primary hepatocytes were stimulated with FBS (10% v/v), high glucose (20 mM) and insulin (50 nM) 3 h and harvested in modified-RIPA buffer; KOH was added to a final concentration of 5M and samples were boiled at 95°C for 2 h. 100% ethanol was added to a final concentration of 80% (v/v) to precipitate glycogen overnight at -20°C. Glycogen was pelleted by centrifugation (15,000x*g*, 15 min 4°C), pellets dried and suspended in 50 mM sodium acetate, 50 mM acetic acid, pH 4.7. Glycogen was digested to glucose by amyloglucosidase (100 U/mL, Sigma #10115), and glucose was quantified as described above. Glycogen concentration was calculated based on a standard curve, normalized to protein content in the original extract.

### Lipogenesis assay

Lipogenesis was stimulated in control or siRNA transfected primary hepatocytes cultured in maintenance medium (low glucose control) or supplemented with 20 mM glucose, 50 nM insulin, 10% FBS (v/v) [43] for 3 days, then fixed in 10% formalin (v/v) for 5 min, washed with PBS and 60% isopropyl alcohol (v/v), and stained with fresh Oil Red O solution for 30 min. Images were captured on a Nikon brightfield microscope with Nikon NIS Elements software. 

### Statistical analyses

Results are reported as mean ±stdev. Differences between groups were considered significant at *p*<0.05 by two-tailed Student’s *t*-test. Data shown are average values of biological replicates within one experiment or averages of independent experiments. Animal data represent biological replicates (independent animals) tested in the same experiment.

## Supporting Information

Figure S1 **NEDD4L regulation in primary hepatocytes.** (A) Quantification of NEDD4L isoform protein abundance from western blots shown in Figures 1C and 2 additional experiments, represented as mean fold induction ±stdev at each time point, *p<0.05 to 0 h control. (B) Time course of *Pepck* and *Pgc1α* mRNA expression in primary hepatocytes treated with glucagon (100 nM); representative of three independent experiments. (C) NEDD4L isoform protein levels in primary hepatocytes treated with indicated doses of glucagon for 3 h (n=3). (D) NEDD4L protein abundance from Figure 1E, normalized to HSP90, expressed mean fold change from *ad* *lib*, ±stdev, *p<0.05 to *ad* *lib*. (E) NEDD4L in cytoplasm/ membrane and nuclear fractions in primary hepatocytes treated with glucagon (100 nM, 3h) (n=3). HSP90 and LAMIN A show relative purity of cytoplasmic and nuclear fractions and equivalent loading. Filled arrowheads, NEDD4L-short; open arrowheads, NEDD4L-long.
(EPS)

Figure S2 **Inhibition of CREB activity in primary hepatocytes.** (A) pXP2-luciferase and pXP2-*Nedd4l-short* luciferase activity in HEK293T cells treated with vehicle (veh, DMSO) or FSK/ IBMX for 6 h, expressed in arbitrary units (A.U., luminescence/ β-galactosidase activity) ±stdev (n=2 experiments in triplicate), **p<0.01 compared to veh. (B) Luciferase assay data shown in Figure 2A expressed in arbitrary units (A.U.) ±stdev (n=3 experiments in triplicate), *p<0.01 between veh and FSK/ IBMX treated; ‡*p*<0.01 to FSK/ IBMX-treated WT; †*p*<0.01 to veh-treated WT. (C) Genomic DNA recovered in chromatin immunoprecipitates (input, IgG IP or CREB IP) from primary mouse hepatocytes as in Figure 2B. The promoter proximal CRE sites of *Nedd4l-short* (CRE2) and *Pepck* and an intragenic region of *Gapdh* were amplified by PCR, analyzed on an agarose gel. (D-F) Primary mouse hepatocytes were infected with adenovirus (Ad) encoding GFP or ACREB, treated with glucagon for the indicated time. (D) Western blots of Flag-ACREB and GAPDH loading control. (E) *Sik1* mRNA normalized to *Gapdh* (mean fold change ±stdev over GFP-infected control). n>3 samples among three independent experiments. (F) Quantification of NEDD4L isoform protein abundance from western blots shown in Figure 2D and two additional experiments, normalized to HSP90, represented as mean fold change of uninfected control, ± stdev. *p< 0.05.
(EPS)

Figure S3 **Lipogenesis in NEDD4L-deficient hepatocytes.** Oil Red O (neutral lipid) staining in (A) untransfected primary mouse hepatocytes cultured in control medium [low glucose (5 mM)] or lipogenic medium [high glucose (25 mM), FBS (10% v/v), insulin (50 nM)] and (B) siRNA-transfected hepatocytes cultured in lipogenic medium. No visible effect of siRNAs (n=2). Bar, 100 µm.
(EPS)

Table S1 **Sequences of oligonucleotide primers and siRNAs.**
(DOCX)
