# Supplementary material for: The Short Isoform of the Ubiquitin Ligase NEDD4L Is a CREB Target Gene in Hepatocytes
Source: PLoS One. 2013 Oct 17;8(10):e78522. doi: 10.1371/journal.pone.0078522 (PMC3798379; doi:10.1371/journal.pone.0078522)
Supplement: Table S1 — Sequences of oligonucleotide primers and siRNAs. (DOCX) [file pone.0078522.s004.docx]

| Description | Forward (5’-3’) | Reverse (5’-3’) |
| --- | --- | --- |
| *mNedd4l-s* | AGAGGGCTCTGCCTGGTGGG | CGAGTGGGCGGAGCAAGGTG |
| *mNedd4l-l* | CGACGCACTTCAGCCAGT | CTTCTTGGCGAGGTCAATTC |
| *mPgc1α* | GGACGGAAGCAATTTTTCAA | TTACCTGCGCAAGCTTCTCT |
| *mPepck* | AGAGTCACCCCTTCCCACTC | CCCTAGCCTGTTCTCTGTGC |
| *mSik1* | ATTGTCCCCATGTTTGTGGT | TACTGCTGCGGTGAGATTTG |
| *mGapdh* | AGGTCGGTGTGAACGGATTTG | TGTAGACCATGTAGTTGAGGTCA |
| *si-Scrambled control* | GAUCAUACGUGCGAUCAGATT | UCUGAUCGCACGUAUGAUCTT |
| *siNedd4l-a* | GAUUUACCUCCAUAUGAAA | UUUCAUAUGGAGGUAAAUC |
| *siNedd4l-b* | UGGAUUUGCCGAACUCUU | AUAGAGUUCGGCAAAUCCA |
| *Nedd4l specific RT* | TACTTCAAATAGGAGCCTGTG |  |
| *Nedd4l-short promoter PCR* | ATAAGCTTTCAGGCAGTCCACACTATGC | TCTCGAGCGACAGCCTGGAACGAGT |
| *Quick change primer (-412~-407 CRE1)* | AAGCAGCCTctagaGTGGAATGTTGTTC | GAACAACATTCCACtctagAGGCTGCTT |
| *Quick change primer (+196~+201 CRE2)* | GCTCTCAGCACcatggGCTCAGCACAG | CTGTGCTGAGCccatgGTGCTGAGAGC |
| *mNedd4l-s CRE2 ChIP primer* | ATGAGCTGTTTCTGTCTTCC | GTAGAGCCCTGCAGAGCTTC |
| *mGapdh ChIP primer* | GAAACCAGGAGCATTTGGAA | CTTGCCTGTAAAGGGAGCAG |
| *mPepck ChIP primer* | CCATGGCTATGATCCAAAGG | CGCCCTCCTTGCTTTAAATA |
